# The Youth Health Care measure-satisfaction, utilization, and needs (YHC-SUN)-development of a self-report version of the Child Health Care (CHC-SUN) proxy-measure

**DOI:** 10.1186/s12913-016-1419-1

**Published:** 2016-05-20

**Authors:** Silke Schmidt, Ute Thyen, Carsten Herrmann-Garitz, Franziska Bomba, Holger Muehlan

**Affiliations:** Department Health and Prevention, Institute of Psychology, Ernst-Moritz-Arndt-University of Greifswald, Robert-Blum-Str. 13, 17489 Greifswald, Germany; Department of Child and Adolescent Medicine, University of Lübeck, Ratzeburger Allee 160, 23538 Lübeck, Germany

**Keywords:** Pediatric health service research, Coordination of care, Transition, Adolescents with chronic conditions, CHC, SUN (= Child Health Care, Satisfaction, Utilization and Needs), YHC, SUN (= Youth Health Care, Satisfaction, Utilization and Needs)

## Abstract

**Background:**

The transition of health care of youth (age 15–25) with chronic conditions requires the assessment of adolescents’ access, use and needs as well as satisfaction with the health services they use. The aim of this study was to test the adolescent adaptation of the parent version “Child Health Care Questionnaire - Satisfaction, Utilization and Needs” (CHC-SUN) concerning its psychometric performance and appropriateness for adolescents and young adults.

**Methods:**

The Youth Health Care Measure (YHC-SUN) was designed to allow self-report of youth and it was pilot-tested in a small sample using cognitive debriefing. A cross-sectional survey in a sample of youth with chronic conditions in the transition period was carried out.

**Results:**

One hundred eighty-two ambulatory care patients with three conditions participated in the survey. The subscales of the section on satisfaction with care showed excellent internal consistencies, uni-dimensionality and fit to the model of the parent version. There was no impact of gender and education on satisfaction with care. Associations with age, diagnosis, experiences with care and health literacy affecting the satisfaction with care indicate discriminatory and content validity.

**Conclusions:**

Potential applications of the new instrument are evaluations of health care services for adolescents and young adults using self-reports and evaluations of transition programs and interventions such as patient education.

## Background

Health service research in adolescents and adulthood has gained more attention during recent years for different reasons: (1) the need for appropriate, acceptable and efficient health services to meet this population’s needs [1, 2] and (2) the rising number of adolescents growing up with chronic conditions and requiring life-long management [3, 4], as well as (3) the notion of including the views and satisfaction of consumers of health care [5]. Self-reports are an integral part of health service research in pediatric care [6, 7]. While in quality of life (QoL) research the inclusion of QoL instruments in children has already developed as a standard approach [8], health service research of children and adolescents with chronic conditions has predominantly included parent or observer reports. An evaluation of interventions targeting youth should include the participants evaluating outcomes of care as their views have been shown to be different from those of their parents [6, 9, 10]. Chronic conditions impact in various ways on the lives of young people, in transition periods in particular when leaving home and engaging in partnerships. Training, education and managing the disease may create an extra burden compared to the usual challenges of growing up [4]. Instruments to measure the health care needs and satisfaction with services are required to capture this population’s perspectives.

The instrument ‘Child Health Care - Satisfaction, Utilization and Needs’ (CHC-SUN) has been developed cross-culturally to evaluate pediatric health care services for children and adolescents with special health care needs from the proxy perspective of parents [11, 12]. With a growing number of youth engaging in patient education programs which support the transition process we saw a need for a corresponding self-report version assessing health care satisfaction, utilization and needs from the perspective of adolescents and young adults [12–14]. While some aspects of health care may be similar across conditions, especially psychosocial care, some aspects of the type and characteristics of a condition can lead to differences in the way health services are delivered. We were interested in the impact of various characteristics of conditions and of different kinds of health care [3]. In particular we assumed that access to a fairly standardized evidence based treatment and a predictable course of the condition (i.e., in type 1 diabetes) vs. treatment with large variation due to lack of evidence and predominance of individual factors (i.e. multiple sclerosis, inflammatory bowel disease) would have an impact on satisfaction with services.

The aim of the current study was to test whether the CHC-SUN [11] can be adapted to an adolescent version (=YHC-SUN) based on focus group research, piloting the instrument and cognitive debriefing as well as finally field testing. The current paper reports on the results of the adaptation. Validity in terms of content validity was assessed using items from part 1 of the questionnaire, addressing receipt of services and settings of care, specific characteristics of the burden of the condition and unmet health needs. We assumed that these factors would be associated with higher or lower satisfaction with care. Given that self-management and patient autonomy are associated with health management competencies we also assumed that higher levels of health literacy would reduce unmet health needs and thus increase satisfaction with care [15, 16].

## Methods

Ethical approval for the study was obtained by the University Medical Center of Greifswald. All procedures performed in studies involving human participants were in accordance with the ethical standards of the institutional and national research committee and with the 1964 Helsinki declaration and its later amendments or comparable ethical standards. This article does not contain any studies with animals performed by any of the authors. Informed consent was obtained from all individual participants included in the study.

### Measures

The instrument CHC-SUN is a measure for health service evaluation from the perspective of parents with chronic conditions. It is based on a conceptual model linking health care needs with processes of care and satisfaction with care [13]. The 57-item-instrument is structured into two modules. Module 1 comprises 30 single items with respect to provision (e.g. frequency of visits), utilization, access problems, and satisfaction with the most common pediatric/adolescent services for chronic health conditions: general practitioner (GP), specialist care, prescribed medicine, and emergency services. Furthermore unmet needs were assessed with respect to 16 services including also the coordination of care. The response categories included whether a service was (a) both needed and fully or partly used, (b) not needed and not used, or (c) needed but not used. We labeled the latter category as “unmet need” from the perspective of the user.

Module 2 is related to processes and satisfaction with care and comprises six scales in the previously published parent report form with Cronbach’s alpha reaching values above .80 for each of the scales, indicating high reliability for all scales of the measure in terms of internal consistency [11]: “diagnosis/information” (Cronbach’s alpha/α = .91), “coordination” (α = .80),” child-centered care” (α = .95), “hospital environment” (α = .84), “doctors’ behavior” (α = .96) and “school services” (α = .89) as well as a single item on “general satisfaction with health care”. Response choices on a five point scale were: not satisfied/partly satisfied/satisfied/very satisfied/extremely satisfied. In a field test in a sample of 795 parents [11] having children with six different types of chronic conditions the overall number of unmet needs inversely correlated with the general-satisfaction-item (*r* = .35, *p* ≤.001). Furthermore, satisfaction with care was positively related to the health-related quality of life of children and of parents. The scale has been validated in several studies, for instance highlighted unmet needs in traumatic brain injuries [17, 18] and epilepsy [19] as well as in rare diseases [20].

For validation purposes, a multidimensional health literacy measure was used with respect to the transition period [21]. The instrument comprises 3 domains: “work-related preparedness” (3 items, α=.72), “condition-related knowledge” (3 items, α=.72) and “health-care competence” (4 items, α=.72). All items can be combined to a global score “transition competencies” (10 items, α=.81).

### Process of instrument adaptation

A qualitative bottom-up-approach was used in order to validate whether the conceptual underpinnings of the instrument were equivalent to the version for parents of children with chronic conditions. Six focus groups and eleven interviews including youth with diabetes, cystic fibrosis as well as chronic inflammatory bowel disease were conducted in order to cover different chronic conditions (*n*=29). The qualitative results showed that adolescents are concerned about the same areas in health care consequences as parents [22]. Furthermore items concerning access and structure of health services were assessed on a qualitative level.

Items of the CHC-SUN were reformulated in first-person answer format. Cognitive debriefing sessions with four adolescents showed that three items related to financial issues (payment for services, and financial burden) had to be eliminated due to a lack of understandability. In order to increase understandability one item was added to check the suitability of questions regarding time of diagnosis. The resulting questionnaire with all item wordings is provided in the supplementary material (Table 8).

### Procedure of recruitment for the field survey of the YHC

Patients aged between 15 and 25 years were contacted at their clinic visit by postal mail or via provision of website access for online assessment in various German regions. Participants were motivated by focusing on the need to let adolescents participate in health service evaluation. If they were interested in the study they received either the postal questionnaire or access to the website. Targeted study participants in terms of inclusion criteria of the sample were adolescents with an ongoing chronic condition aged between 15 to 25 years. The resulting sample provides the first data base for the self-report measure of the YHC-SUN. Preliminary studies investigating similar samples (e.g. the European DISABKIDS study on quality of life in children and adolescents with chronic conditions conducted between 2002 and 2005) only applied a proxy-report version of the CHC-SUN.

### Data analysis of the field survey of the YHC SUN

Data analysis comprised descriptive analysis of all items including the investigation of missing data and response patterns and concerning the association between sociodemographic and diagnostic information on the one hand and items assessing use of and access to health services on the other hand. Items and composite scales were evaluated regarding missing data rate and basic descriptive distribution indicators. Split-half reliability (Guttman’s coefficient) and homogeneity (Cronbach’s alpha coefficient) of the scales were identified, and item internal reliability (corrected item-total correlation) was investigated. Confirmatory factor analysis on the satisfaction scales was conducted in order to test whether the model of the parent version fits to the youth version. In addition to classical psychometric analysis, Rasch analysis was performed applying the partial credit model [23], an extension of the original Rasch-model to ordinal variables. Rasch-analysis was used to specifically cover possible misfit on item level. Item (mis)fit was detected by Q-index statistics and threshold ordering estimation.

Basic descriptive and statistical analyses of the data were performed using IBM SPSS Statistics 21.0 software. AMOS 19 software was used to conduct confirmatory factor analysis. Rasch model analysis was performed using WINMIRA software.

## Results

### Sample characteristics

A total of 182 youth aged 15 to 25 years (m=19.4 years) with a variety of chronic conditions participated in the study with a higher rate of females participating (Tab. 1). Among all respondents about 40 % attended school, 14 % had started vocational training and 26 % higher education. Nearly 10 % were already in employment and 3 % reported to be unemployed.

**Table 1:** Sample characteristics (*N*=182)^a^

Characteristics		n^b^	%^c^
Age groups (mean: 19.4, Range: 15–25)	15–17 years	42	23.1
	18–21 years	93	51.1
	22–25 years	46	25.3
Gender	Female	129	70.9
	Male	53	29.1
Current occupation	Pupil	74	40.7
	Vocational Training	26	14.3
	Student	48	26.4
	Unemployed	6	3.3
	Other	10	5.5
	Employed	18	9.9
Highest level of education	Still attending school	59	32.4
	Certificate of special school	3	1.6
	Certificate of secondary education	13	7.1
	General certificate of secondary education	41	22.5
	Final secondary-school examinations	59	32.4
	Other certificate	6	3.3
	Finished school without school leaving certificate	1	0.5
Treating physician	Pediatrician	61	33.5
	Practitioner for adults	119	65.4
Receiving care by a specialist for the condition	Yes	156	85.7
	No	26	14.3

Notes: ^a^Figures refer to numbers and percent unless otherwise indicated. ^b^Difference between numbers reported for each characteristic and total sample (*N*=182) indicate missing data. ^c^Percent are reported as related to valid cases only

The sample included a small number of students attending special schools (*n*=7.4 %) and students with the lowest level of school (*n*=15, 8.2 %), indicating that the questionnaire was adequate for respondents with low reading level. The subsample responding via paper-pencil questionnaires in comparison to the one filling in online questionnaires did not differ with respect to gender, education and the main conditions, however, slightly in relation to age, with young adults being overrepresented in the online sample compared to more adolescents in the paper-pencil version (*p*=.05).

We formed the following sub-groups according to the characteristics of the condition and burden of care (Tab. 2). The largest groups included 45 youth with type 1 diabetes and 33 youth with multiple sclerosis, respectively. The majority of the 28 participants with “chronic conditions associated with gastrointestinal symptoms” had chronic inflammatory bowel disease, of the 19 participants with arthritis the majority suffered juvenile rheumatoid arthritis. Among the group with “pulmonary conditions” (*n*=13) were mainly youth with cystic fibrosis and in the group with “chronic skin conditions” (*n*= 14) youth with atopic dermatitis. The group “other rare conditions” (*n*=30) included mainly congenital and metabolic diseases.

**Table 2:** Characteristics of condition groups (*N*=182)^a^

Diagnoses	Age mean	Age SD	Age range	Further information
Diabetes (*n*=45)	18.5	2.4	16–25	- mean duration of illness 7.7 [SD 5.2] ys
				- 20 (44 %) in pediatric specialist care
				- those who were in adult specialist services had made the transition on average 3.6 years ago.
Multiple sclerosis (*n*=33)	20.5	2.3	16–25	- mean duration of illness 2.0 [SD 1.5] ys
				- 2 (6 %) in pediatric specialist care
				- those who were in adult specialist services had made the transition on average 6.1 years ago
Chronic conditions associated with gastrointestinal symptoms (*n*=28)	20.3	1.8	16–25	- mean duration of illness 4.0 [SD 3.0] ys
				- 4 (14 %) in pediatric specialist care
				- those who were in adult specialist services had made the transition on average 4.0 years ago
Arthritis (*n*=19)	17.7	1.5	16–21	- mean duration of illness 7.4 [SD 5.5] ys
				- 11 (58 %) in pediatric specialist care
				- those who were in adult specialist services had made the transition on average 1.7 years ago
Chronic skin conditions (*n*=14)	19.6	2.7	17–25	- mean duration of illness 13.2 [SD 6.8] ys
				- 1 (7 %) in pediatric specialist care
				- those who were in adult specialist services had made the transition 5.8 years ago
Pulmonary conditions (*n*=13)	19.1	2.6	16–25	- mean duration of illness 17.0 [SD 4.3] ys
				- 9 (69 %) in pediatric specialist care
				- those who were in adult specialist services had made the transition on average 1.3 years ago
Other rare chronic conditions (*n*=30)	19.5	3.2	15–25	- mean duration of illness 12.9 [SD 9.4] ys
				- 14 (47 %) in pediatric specialist care
				- those who were in adult specialist services had made the transition on average 5.9 years ago

### Receipt of services (Module 1)

In module 1 of the questionnaire assessing receipt of services, access to care, satisfaction with specialist care and unmet needs *missing data* ranged between 0.5 % (*n*=1) and 6.6 % (*n*=12) across items, with varying highest frequencies of missing data per item depending on condition (diabetes: *n*=5; pulmonary conditions *n*=1; all other conditions *n*=2 each). Source of health care services, access to services and self-reported unmet health needs revealed an extremely heterogeneous and complex picture across conditions.

We asked participants about the *source of care* for their chronic condition. A majority of all respondents (85.7 %) reported to receive *specialist care* for their condition. Younger adolescents (age 15–17) were more likely to report a pediatrician (68.3 %) compared to adolescents (age 18–21; 32.2 %) and young adults (age 22–26; 8.7 %). Type of condition and source of specialist care were highly associated (*p*<.001): the rate of patients with specialists caring for adults were more than 90 % in multiple sclerosis and chronic skin diseases, approximately 50 % in diabetes and other rare conditions and about 30 % in pulmonary conditions. Half of all adolescents receiving specialist care reported no difficulties in access to specialist care (55 %), 34.8 % reported some difficulties and 9.7 % great difficulties. Overall satisfaction with specialist care was moderate: only 53.2 % reported high satisfaction, 22.4 % were satisfied, whereas 24.4 % were partly satisfied or fully dissatisfied.

When probing whether respondents had a *primary care physician* (pediatrician or general practitioner), 23.1 % reported not to have such care, respective proportions were similar across all condition groups. Youth who reported to attend a specialist for adult care for their condition were less likely to have a primary care physician (74 %) compared to those who attended a pediatric specialist (85 %).

Almost all (91 %) participants reported to have received *prescriptions for medication* and of those only 7 % reported major difficulties in access to needed medication. Satisfaction with the medication was higher in adolescents with diabetes compared to all other conditions. One quarter (24.9 %) of all respondents had received *emergency care*. Youth with multiple sclerosis were twice as likely to have accessed emergency care (45.5 %), those with arthritis (5.9 %) and atopic dermatitis (13.3 %) were least likely. In diabetes and multiple sclerosis, half of respondents reported difficulties in getting emergency services when needed. Satisfaction with emergency care was extremely heterogeneous and was lowest in adolescents with multiple sclerosis.

*Unmet health care needs* were most frequently reported in terms of dietary counseling (23.6 %), health education (22.5 %) and psychological counseling (21.4 %) across all conditions (see Table 3). Participants reported very few unmet needs in the area of supply with medical equipment or physical aids, home nursing services or respite care. Some services had high relevance to some conditions but not to others: whereas only 11 % of adolescents with diabetes reported unmet needs in the area of patient education, the respective proportion was 30 % in multiple sclerosis, 36 % in conditions associated with gastrointestinal symptoms*,* 53 % in chronic skin conditions*.* While most youth with arthritis received physical and/or occupational therapies and did not report unmet needs in this area, 21 % of those with multiple sclerosis reported unmet needs in physical therapy and 18 % in occupational therapy. Youth with conditions associated with gastrointestinal symptoms reported highest rates of unmet needs in the area of school or work related counseling or coordination (36 %). The total score of unmet needs was significantly higher in those who did not have a primary care physician (primary care: 3.73; no primary care: 3.55) and those who reported problems in access to specialist care (extreme difficulties: 7.25; strong difficulties: 5.90; difficulties: 4.50; some difficulties: 2.92; no difficulties: 2.58; see Table 4).

**Table 3:** Unmet health care needs by diagnosis in order of total frequency (response choice option “not received, but needed”)

		Diagnosis						
Unmet needs	Total	Diabetes	Multiple sclerosis	Conditions with gastro-intestinal symptoms	Arthritis	Chronic skin conditions	Pulmonary conditions	Other conditions
	%	%	%	%	%	%	%	%
Dietary counseling	23.6	11.1	27.3	46.4	15.8	33.3	15.4	20.7
Health education	22.5	11.1	30.3	35.7	0.0	53.3	23.1	17.2
Psychological counseling	21.4	15.6	27.3	39.3	10.5	26.7	23.1	10.3
Health services at school/at work	15.9	6.7	27.3	35.7	5.3	13.3	15.4	6.9
Self-help groups	14.8	6.7	18.2	39.3	0.0	20.0	30.8	0.0
Coordination of services	13.2	2.2	12.1	39.3	5.3	20.0	23.1	3.4
Physiotherapy	12.1	6.7	21.2	17.9	0.0	13.3	15.4	10.3
Rehabilitation services	11.5	2.2	21.2	17.9	5.3	6.7	23.1	10.3
Occupational therapy	7.7	0.0	18.2	14.3	5.3	0.0	15.4	3.4
Telephone counseling	7.7	0.0	6.1	21.4	5.3	20.0	0.0	6.9
Social worker services	7.1	2.2	6.1	14.3	5.3	6.7	15.4	6.9
Short-term respite care	3.8	0.0	3.0	3.6	0.0	13.3	7.7	6.9
Speech therapy	3.3	0.0	6.1	3.6	0.0	0.0	15.4	3.4
Supply with physical aids	2.7	0.0	9.1	0.0	10.5	0.0	0.0	0.0
Stomatherapy	2.7	0.0	6.1	3.6	5.3	0.0	7.7	0.0
Nursing services at home	2.2	0.0	3.0	3.6	0.0	0.0	7.7	3.4
Supply with medical equipment	1.1	0.0	3.0	0.0	0.0	0.0	7.7	0.0

Note: The response choice option “not received but needed” implies that a person feels that he needs a service but does not receive it

**Table 4:** Unmet health care needs by source of care in order of total frequency (response choice option “not received, but needed”)

Unmet needs	Total	Attending pediatric specialist	Attending adult specialist	No specialist	Having a primary care physician	Having no primary care physician
	%	%	%	%	%	%
Dietary counseling	23.6	5.7	31.7	30.8	25.0	19.0
Health education	22.5	5.7	29.7	26.9	17.9	38.1
Psychological counseling	21.4	5.7	29.7	23.1	20.0	26.2
Health services at school/at work	15.9	7.5	18.8	23.1	12.1	28.6
Self-help groups	14.8	7.5	18.8	15.4	17.1	7.1
Coordination of services	13.2	9.4	12.9	23.1	12.1	16.7
Physiotherapy	12.1	5.7	15.8	11.5	12.1	11.9
Rehabilitation services	11.5	9.4	14.9	3.8	11.4	11.9
Occupational therapy	7.7	3.8	7.9	15.4	8.6	4.8
Telephone counseling	7.7	0.0	10.9	7.7	7.1	9.5
Social worker services	7.1	7.5	7.9	3.8	7.9	4.8
Short-term respite care	3.8	1.9	5.0	3.8	3.6	4.8
Speech therapy	3.3	1.9	4.0	3.8	2.1	7.1
Supply with physical aids	2.7	0.0	4.0	3.8	2.9	2.4
Stoma therapy	2.7	1.9	3.0	3.8	2.9	2.4
Nursing services at home	2.2	1.9	2.0	3.8	2.1	2.4
Supply with medical equipment	1.1	0.0	2.0	0.0	1.4	0.0

Note: The response choice option “not received but needed” implies that a person feels that he needs a service but does not receive it

### Satisfaction with care (Module 2)

In module 2 of the questionnaire *missing data* ranged between 1.1 % (*n*=2) and 16.0 % (*n*=20) across items, with varying highest frequencies of missing data per item depending on condition (diabetes: *n*=9; multiple sclerosis & chronic skin conditions *n*=5 each; arthritis & other rare chronic conditions *n*=4 each; pulmonary conditions & chronic conditions associated with gastrointestinal symptoms *n*=3 each). The scales were labelled (1) “diagnosis/information”, (2) “coordination”, (3) “patient-centred care”, (4) “clinic environment”, (5) “doctors’ behaviour”, and (6) “school-related services”; in addition there was a single item called “general satisfaction”. We found only higher levels of missing data in the “school-related services” scale. The scale has proven as extremely valid [11], however is felt to be not applicable to many adolescents.

The internal consistencies of the six scales were high (Cronbach’s alpha ranging between .75 and .96). Psychometric results of response scales are distributed in Table 5 indicating good scale performance.

**Table 5:** Descriptive and psychometric analysis of the YHC-SUN Module II satisfaction domains

Domain	No. of items	r _II_	r _IT_	r ^2^	G	α
Diagnosis/Information	5	.33–.74	.45–.76	.31–.67	.86	.85
Coordination	3	.40–.62	.50–.66	.26–.46	.75	.75
Patient-centred care	4	.81–.88	.88–.92	.78–.84	.96	.96
Hospital environment	5	.20–.73	.47–.73	.28–61	.86	.80
Doctors’ behaviour	5	.58–.81	.75–.87	.62–.77	.91	.93
School related services	3	.68–.80	.74–.84	.56–.71	.79	.89
General satisfaction^a^	1	--	--	--	--	--

Notes: ^a^ Single item measure, psychometric analysis not applicable; r _IT_ = item-total-correlation (part-whole corrected); *r* ^*2*^ = squared multiple correlation of items; r_II_= inter-item-correlation; G=Guttman’s split-half reliability; α = Cronbach’s alpha

Accordingly, Rasch analysis indicated ANOVA reliabilities ranging between .82 and .93 and Andrich’s reliabilities ranging between .77 and .93 (see Table 6). No item misfit has been detected and thresholds are ordered for all item categories along the latent traits of the scales, except for one item assigned to the “school related services” scale, with two thresholds marginally misordered (−.004).

**Table 6:** Rasch analysis of the YHC-SUN Module II satisfaction domains

Domain	No. of items	ANOVA reliability	Andrich’s reliability	Q-index	Item misfit	Misordered thresholds
Diagnosis/Information	5	.85	.82	.08–.15	0	0
Coordination	3	.87	.85	.02–.03	0	0
Patient-centred care	4	.93	.93	.01–.02	0	0
Clinic environment	5	.82	.77	.05–.14	0	0
Doctor’s behaviour	5	.90	.89	.03–.06	0	0
School related services	3	.85	.82	.02–.05	0	1
General satisfaction^a^	1	---	---		---	---

Notes: ^a^ Single item measure, Rasch analysis not applicable

A confirmatory factor analysis (CFA) was conducted in order to test whether the scale structure assumed in the Module 2 of the CHC-SUN can be confirmed in the present sample. Despite of the excellent performance of each single domain, the CFA did not show satisfactory fit due to significant correlation coefficients between the scale scores ranging from .20 to .87.

The relationship of health literacy and satisfaction with care showed diverging associations: the subscale “knowledge with respect to health” was positively correlated with all CHC-SUN satisfaction scales; correlation with general satisfaction was *r*=.34. Higher knowledge was marginally associated with higher satisfaction with respect to provision of school services (*r*=.21). The “transition competencies” subscale was not related to any of the satisfaction scales.

In *multivariate covariance analysis* mean scores of all six satisfaction scales did not differ between females and males. In general, younger adolescents showed higher satisfaction than older adolescents/young adults accordingly, especially for satisfaction with information about the diagnosis and with doctors’ behavior (as well as global satisfaction; see Table 7). For all scales except the school related services subscale we also found significant differences for source of care after controlling for age group: respondents in pediatric care showed higher satisfaction compared to those in adult care.

**Table 7:** Means of satisfaction scores in age groups and diagnostic groups

		Diagnosis/information	Coordination	Patient-centred care	Clinic environment	Doctors’ behaviour	School related services	General satisfaction
		Means (SD)						
Age group	15–17 years	3.27 (1.03)	3.20 (0.97)	3.61 (1.24)	3.04 (0.77)	3.71 (1.01)	2.39 (0.94)	3.53 (0.92)
	18–21 years	2.94 (1,00)	2.80 (0.95)	3.11 (1.24)	2.90 (0.90)	3.26 (1.11)	2.52 (1.15)	3.19 (0.91)
	22–25 years	2.62 (0.97)	2.76 (1.04)	3.12 (1.32)	2.94 (1.07)	3.04 (1.06)	2.39 (1.06)	2.87 (1.10)
Diagnosis	Diabetes	3.50 (0.87)	3.27 (0.75)	3.75 (1.11)	3.30 (0.75)	3.78 (0.90)	2.35 (1.10)	3.62 (0.83)
	Multiple sclerosis	2.59 (0.99)	2.74 (1.00)	2.98 (1.36)	2.92 (1.07)	3.06 (1.24)	2.71 (1.27)	2.93 (1.03)
	Chronic conditions associated with gastrointestinal symptoms	2.66 (0.99)	2.62 (1.00)	2.78 (1.14)	2.89 (1.09)	3.05 (1.12)	2.33 (1.00)	2.85 (1.01)
	Arthritis	3.36 (0.97)	3.28 (0.90)	3.50 (1.16)	3.09 (0.87)	3.81 (0.90)	2.41 (0.88)	3.53 (1.01)
	Chronic skin conditions	2.30 (0.87)	1.92 (0.68)	2.73 (1.42)	2.40 (0.61)	2.67 (1.13)	2.03 (0.96)	2.73 (0.90)
	Pulmonary conditions	2.85 (1.22)	2.88 (1.17)	3.25 (1.59)	2.81 (0.91)	3.35 (1.25)	2.75 (1.33)	3.58 (0.90)
	Other rare chronic conditions	2.78 (0.86)	2.80 (1.04)	3.10 (1.11)	2.73 (0.73)	3.08 (0.99)	2.49 (0.93)	2.88 (0.89)

There were significant differences in satisfaction with care between diagnostic groups (except for “school-related services”), most pronounced in the “information/diagnosis”, “coordination”, “patient-centered care” and “doctors’ behavior” scale (see Tab. 7).

We found significant associations with general satisfaction with the specialist (item from module 1) in all scales except “school-related services” Tab.8. Similarly, we found moderate negative correlations of the six satisfaction scales with the sum of unmet needs. Unmet needs are also associated with low general satisfaction with services (*r*=−.44).

**Table 8:** Associations of dimensions of satisfaction and overall satisfaction with specialist care and unmet needs

CHC-SUN domains		
Satisfaction domains (Module 2)	Satisfaction with the specialist (Module 1 item)	Sum score of unmet needs (Module 1)
Diagnosis/Information	.53	-.53
Coordination	.62	-.58
Patient-centered care	.70	-.51
Clinic environment	.55	-.37
Doctors’ behaviour	.72	-.48
School related services	.23	-.16
General satisfaction^a^	.49	-.44

Notes: ^a^ Single item measure. All values are Spearman rank correlation coefficients

## Discussion

There has been a long tradition of integrating children and adolescents in questionnaire development in the area of quality of life [24], however, approaches of participation of adolescents in health service evaluation have often been investigated in specific health care areas and projects and not to date from a generic perspective. The adolescent adaptation of the health service research instrument YHC-SUN in youth has been shown to be reliable and valid across a range of conditions for the scales measuring satisfaction with care (module 2). Participants with various educational levels were able to participate, the proportion of missing responses was on average low. The type of the condition and gender did not affect the acceptability and the performance of the instrument. The psychometric properties of the instrument are very good using both criteria of classical and probabilistic psychometric test theory [25]. All reliability coefficients indicate sufficient to excellent reliability of the sub-scales. For most sub-scales, items represent a broader range of item-total correlations with just a few items reaching low values. With respect to Rasch analysis, no item misfit could be identified. Except for 1 out of 100 thresholds, ordering of thresholds is in accordance with model assumptions. Validity in terms of differentiating between known groups could be demonstrated. Methodologically, there is potential for a shortened version of satisfaction domains, due to significant intercorrelation coefficients [26] of scales. Considering the need for short assessment in health care research, the development of such a brief measure is under way.

The instrument shows also differential sensitivity of items and scales regarding the receipt of health care services in the transition period. The pattern of transition processes showed substantial differences across conditions as already observed in the CHC-SUN, however higher effects of age as in the parent version for younger children with chronic conditions [11]. The higher satisfaction values of younger adolescents which decrease during the age of transition at early adulthood demonstrate the importance to provide developmentally appropriate healthcare across the age range of young people [27]. The decrease in satisfaction may in part be due to the fact that adolescents achieve growing understanding and cognitive skill, begin to frame their independent views about their social environment and are encouraged to develop their own points of views. This development should be acknowledged and welcomed and must be taken into account in preparing adolescents for transition [2, 28]. Although we do not have data to support the interpretation that quality of care decreases as adolescents get older, a recent review reported that despite some advances in health care for adolescents in some areas, young adulthood continues to entail greater risk and worse outcomes than adolescence [29]. However, we did find an effect of source of care after taking age into account. Participants in pediatric care report higher satisfaction compared to those in adult specialist services and report much less unmet needs. We also found a confounding factor of condition group and source of care as the majority of patients in multiple sclerosis, gastrointestinal chronic conditions and skin disease are being treated by adult specialists irrespective of age – in all these groups satisfaction with services is lower compared to conditions remaining in pediatric care. We were able to show that satisfaction with health care is highest in conditions with a homogenous profile and available standard, evidence based guidelines such as type 1 diabetes, juvenile arthritis and cystic fibrosis (majority of participants in the pulmonary group) and easy access to clinics for outpatient adolescents. In childhood onset type 1 *diabetes,* care is typically transitioned around age 16 in a fairly standardized manner both in pediatric and in adult services [30]. Access to specialized care, medication and patient education programs is easier compared to other conditions. Unmet needs were low in this group and satisfaction with services high. Participants with cystic fibrosis remain mainly in pediatric care even as young adults (90 % in our sample); adult pneumologists usually do not provide services as survival in recent decades was limited to childhood and adolescence [31]. The same trend was true for participants with chronic arthritis. Multiple sclerosis and inflammatory bowel disease have an onset of illness in later adolescence and a higher prevalence in the adult population (multiple) and therefore they are more likely to be treated in adult services compared to conditions specific to childhood and low prevalence in the adult population. In chronic skin diseases, particularly atopic dermatitis, treatment often exceeds the therapeutic options of the primary care physician and with a lack of pediatric dermatologists parents turn to adult services early on; satisfaction with services in this group is lowest. We believe that the organization of health care for children and adolescents reaching into young adulthood should center on the needs of this population including their families. While there are specific condition-related needs and requirements for high quality medical care, many issues may be jointly organized by outpatient departments, such as psychosocial care, access to peer and support groups, transitional care, case management and information policies.

As expected, we were able to show that higher levels of health literacy reduce unmet health needs and thus increase satisfaction with care.

Unmet needs contribute to low general satisfaction with services; they are more prevalent in those groups receiving adult specialist care and thus adult care is associated with lower satisfaction. However, since certain conditions are being treated by adult specialists even during childhood we are unable to disentangle the effects of services available for a certain condition and source of care. For example, patient education programs are standard care in diabetes and most children, adolescents and their parents participate at least in one, often two to three group education programs; in this study only 11 % in this group reported unmet needs. Restrictions in the availability and the funding of patient education programs result in much higher rates of unmet needs in participants with multiple sclerosis (30 %), chronic gastrointestinal conditions (36 %) and skin disease (53 %). We assume that access to patient education programs would increase health literacy, self-efficacy and thus access to care in all children and adolescents with chronic conditions [2]. Based on such experiences transition into adult care or acceptance of adult care may be easier compared to a relatively unprepared transition [32]. To summarize, findings highlight the necessity to include adolescents’ health service evaluation in the transition period [33].

## Conclusions

Adolescents must be encouraged to communicate their needs and indicate to which extent their expectations are being met. The newly developed instrument YHC-SUN-self report will support health policy makers and physicians to evaluate young peoples’ perspectives. The decrease in satisfaction with services as adolescents get older and are more likely to receive care in adult services highlight areas with potential for improvement, specifically with respect to the coordination of care and information given about treatment and diagnosis as well as general issues of doctors’ behavior.

### Ethics approval and consent to participate

Ethical approval for the study was obtained by the University Medical Center of Greifswald. All procedures performed in studies involving human participants were in accordance with the ethical standards of the institutional and national research committee and with the 1964 Helsinki declaration and its later amendments or comparable ethical standards. This article does not contain any studies with animals performed by any of the authors. Informed consent was obtained from all individual participants included in the study.

### Consent for publication

Not applicable.

### Availability of data and materials

Due to data protection issues, the data cannot be shared.

## Abbreviations

α: Cronbach’s alpha coefficient
CFA: Confirmatory factor analysis
CHC-SUN: Child Health Care - Satisfaction, Utilization and Needs
F: *F*-value
GP: General practitioner
m: Mean
n: Number
p: *P*-value
QoL: Quality of life
r: Pearson product–moment correlation coefficient
YHC-SUN: Youth Health Care- Satisfaction, Utilization and Needs
